# An efficient Au catalyst supported on hollow carbon spheres for acetylene hydrochlorination

**DOI:** 10.1039/c9ra06989e

**Published:** 2019-10-07

**Authors:** Lihua Kang, Mingyuan Zhu

**Affiliations:** College of Chemistry and Chemical Engineering of Yantai University Yantai Shandong 264005 PR China zhuminyuan@shzu.edu.cn +86-993-2057277; School of Chemistry and Chemical Engineering of Shihezi University Shihezi Xinjiang 832000 PR China

## Abstract

Mesoporous hollow carbon spheres (HCSs) were prepared using SiO_2_ spheres as a hard template, and Au nanoparticles were then synthesized using NaBH_4_ as a reducing agent on the surface of the HCS support. Transmission electron microscopy characterization indicated that Au nanoparticles were much smaller on the HCS support than those on the active carbon (AC) support. HCl-TPD showed that the Au/HCS catalyst displayed a more active site than on Au/AC. The resulting Au/HCS catalyst showed excellent catalytic activity and stability for acetylene hydrochlorination. Acetylene conversion of Au/HCS can be maintained above 92% even after 500 h of lifetime. The excellent catalytic performance of Au/HCS was attributed to the presence of the HCS support, which limited the aggregation of Au nanoparticles.

## Abstract 2

Mesoporous hollow carbon spheres (HCS) were prepared and applied as the support of Au catalyst for acetylene hydrochlorination. Au/HCS exhibited excellent stability for acetylene hydrochlorination.

## Graphical abstract

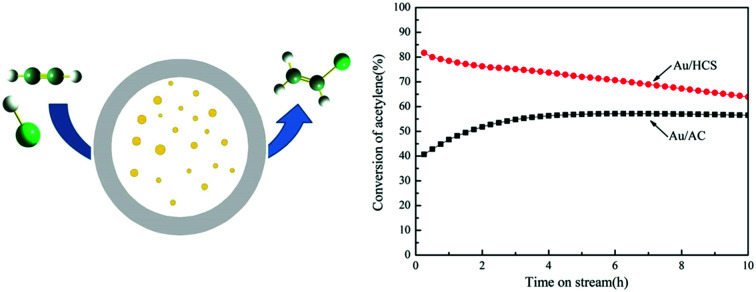

## 1. Introduction

Polyvinyl chloride is mainly produced by polymerization of vinyl chloride monomer (VCM). Acetylene hydrochlorination is an important industrial process in the manufacture of VCM in coal abundant regions, especially in China. Mercuric chloride (HgCl_2_) is commonly used as a catalyst for the industrial processing of acetylene hydrochlorination,^1^ but it is highly toxic and may lead to serious environmental pollution problems. Therefore, the use of mercury-free catalysts has attracted great attention in recent years.

In 1985, Hutchings *et al.* evaluated the catalytic performance of more than 20 types of metal chlorides for acetylene hydrochlorination.^2^ They found that their catalytic activity correlated with the standard electrode potential of the cations present. In 2015, active carbon (AC) supported Au catalysts with ultra-low metal loading and long lifetimes for acetylene hydrochlorination were synthesized. The active sites of the Au catalyst were proposed to be related to the highly dispersed Au that cycles between the Au(i)/Au(iii) redox couples with a S-containing ligand as the soft donor atoms.^3^ Later, Hutchings's group found that single-site cationic Au entities exhibited activity that correlated with the ratio of Au(i)/Au(iii) in the catalyst.^4^ Although the deactivation of the catalyst might be associated with the formation of metallic Au particles,^5^ metallic Au played a role in the catalysis of acetylene hydrochlorination, when the size of Au nanoparticles was controlled to a very small scale. Perez-Ramirez *et al.* found that Au single atoms hosted on N-doped carbon-derived by the controlled pyrolysis of polyaniline exhibited remarkable stability for acetylene hydrochlorination.^6^ In our previous work, we reported that metallic Au nanoparticles exhibited considerable catalytic activity for acetylene hydrochlorination, and the aggregation of Au nanoparticles was another reason for the deactivation of the Au-based catalyst in addition to the reduction of Au(iii) or Au(i).^7^ Therefore, this study used metallic Au as an active site for acetylene hydrochlorination. The use of nitrogen-doped carbon may be a good strategy for supports to inhibit the aggregation of Au nanoparticles and promote support stability for acetylene hydrochlorination.^8,9^ Recently, Yuan *et al.* reported that metallic Au(0) was directly involved in the catalysis of acetylene hydrochlorination due to the strong mediating properties of Ce(iv)/Ce(iii) with one-electron complementary redox coupling reactions. Thus, the ceria promotion to Au catalysts gives enhanced activity and stability.^10^ The reduction of oxidative Au cations and aggregation of Au nanoparticles cannot be avoided under the acetylene hydrochlorination reaction conditions-especially in long-term testing. Thus, another good strategy to improve catalyst stability may be the use of an appropriate carbon support for Au catalysts. We found that larger AC pore sizes increased the speed of the reaction and efficiently prohibited carbon deposition.^11^ Li *et al.*^12^ proposed that some of the oxygenated groups on the surface of AC could improve the catalytic activity and stability of Au catalysts.

HCS has been well reported as catalyst supports due to their high surface area, large pore size, and abundant oxygenated groups;^13^ the hollow structure functions act as a barrier to prevent encapsulated Au nano-particles from aggregation or leaching.^14^ Therefore, we inferred that Au aggregation could also be restricted during acetylene hydrochlorination, and an Au catalyst with excellent stability could be obtained by depositing Au nanoparticles on HCS support.

## 2. Materials and methods

### 2.1 Materials

Tetraethyl orthosilicate (TEOS), cetyltrimethylammonium bromide (CTAB), resorcinol, formalin, activated carbon (coconut carbon, 40–60 mesh, marked as AC), HAuCl_4_·4H_2_O (with 47.8% Au content), NaBH_4_, C_2_H_2_ (gas, 99%), and HCl (gas, 99%) were used here.

### 2.2 Catalyst preparation

#### 2.2.1 Preparation of the SiO_2_ template

SiO_2_ sphere synthesis was performed using a modified Stöber reaction.^15^ Water (25 mL), 63 mL of 2-propanol, and 13 mL of ammonia (27%, aqueous solution) were mixed and heated to 35 °C in a water bath. Next, 0.6 mL of TEOS was added dropwise under vigorous stirring for 30 min to generate the silica seeds. TEOS (5 mL) was then added dropwise. The reaction mixture was held constant at 35 °C for 4 h. The SiO_2_ spheres were separated by centrifugation, washed with ethanol four times, then air dried.

#### 2.2.2 Preparation of mesoporous hollow carbon spheres

Next, 0.8 g of as-obtained SiO_2_ spheres was uniformly dispersed in 70 mL of deionized water by ultrasonication followed by 2.3 g of CTAB, 0.35 g of resorcinol, 28 mL of ethanol, and 0.1 mL of ammonia. The mixture was heated to 35 °C in a water bath and stirred for 30 min to form a homogeneous dispersion. Formalin was then added to the mixture under stirring. The mixture was cooled to room temperature after 6 h and aged at room temperature overnight without stirring. The resulting silica@resorcinol-formaldehyde (SiO_2_@RF) was collected by centrifugation, washed with water and ethanol several times, and dried. The SiO_2_@RF product was then heated under a nitrogen atmosphere at 5 °C min^−1^ from room temperature to 150 °C and kept at this temperature for 1 h. The temperature was then increased by 5 °C min^−1^ to 800 °C and held for 2 h. The SiO_2_@C product was then treated with a 10% HF solution to remove the silica and generate the hollow carbon nanoparticles.

#### 2.2.3 The preparation of Au/HCS catalyst

The Au/HCS catalyst was prepared using NaBH_4_ reduction methods.^8^ About 1.0 g of the HCS was dispersed in 100 mL water under sonication for 30 min. A solution of HAuCl_4_·4H_2_O (2.1 mL, 1 g/100 mL) was added dropwise to the HCS slurry, and then NaBH_4_ (21 mL, 0.1 M) was added to this suspension under sonication and stirred for 30 min. The resulting mixture was vigorous stirred for 24 h at room temperature. Finally, the solution was filtered, washed several times with distilled water, dried in a vacuum at 60 °C, and named as the Au/HCS catalyst. For comparison, an Au/AC catalyst was prepared with active carbon as support for comparison. The theoretical loadings of Au metal were 1% in those two catalysts.

### 2.3 Catalyst characterization

Surface area analysis was carried out by N_2_ adsorption–desorption at 77 K on a Micromeritics ASAP 2020 instrument. Samples were degassed at 423 K for 15 h before analysis. The X-ray diffraction (XRD) data were collected using a Bruker D8 advanced X-ray diffractometer with Cu-Kα irradiation (*λ* = 1.5406 Å) at 40 kV and 40 mA in the scanning range of 2*θ* between 10° and 90°. The transmission electron microscopy (TEM) used a JEM 2010 electron microscope at the accelerating voltage of 200 kV, a line resolution of 0.14 nm, and a point resolution of 0.23 nm. X-ray photoelectron spectroscopy (XPS) measurements were recorded in an Axis Ultra spectrometer with a monochromatized Al-Kα X-ray source (225 W). The temperature programmed decomposition (TPD) was analyzed using an Auto Chem 2720 instrument with a TCD detector. The samples were pretreated in a hydrogen chloride atmosphere at a reactive temperature of 180 °C for 4 h. High-purity N_2_ (40 mL min^−1^) was then passed through the sample at 100 °C for 20 min. The sample was heated at 10 °C min^−1^ from 50 °C to 800 °C during data collection. Thermogravimetric analysis (TGA) used a Netzsch STA-449 F3 Jupiter (Germany) analyzer with oxygen atmosphere, flow rate of 30 mL min^−1^, and a heating rate of 10 K min^−1^.

### 2.4 Catalyst evaluation

Catalytic performance during acetylene hydrochlorination was tested using a fixed-bed reactor. Nitrogen (50 mL min^−1^) was used to remove air and moisture from the reactor system before the start of the reaction. Hydrogen chloride gas at a flow rate of 20 mL min^−1^ was passed through the reactor to activate the catalyst (2 mL). The reactor was heated to 180 °C, and the hydrogen chloride (33.35 mL min^−1^) and acetylene (29 mL min^−1^) were passed at a gas hourly space velocity (GHSV) of 870 h^−1^. The reaction products were detected with a GC-2014C gas chromatograph (Shimadzu).

## 3. Results and discussion

**Fig. 1:**
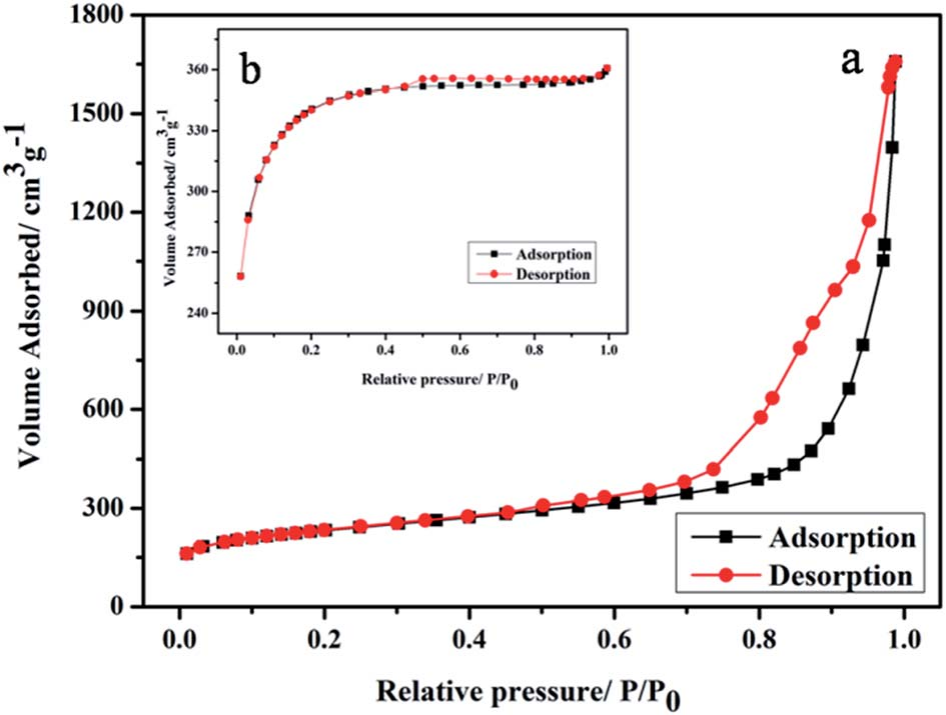
Nitrogen adsorption–desorption isotherms of (a) HCS and (b) AC.

BET was used to analyze the structure of the HCS. Fig. 1a shows that the nitrogen adsorption isotherms of the HCS were consistent with the type IV curve, which indicated that the pore structures of HCS were mainly mesoporous.^16^ The surface area of HCS was calculated to be 762 m^2^ g^−1^, which is lower than that of AC (1033 m^2^ g^−1^) used as the support of Au nanoparticles in the following experiment for comparison.

**Fig. 2:**
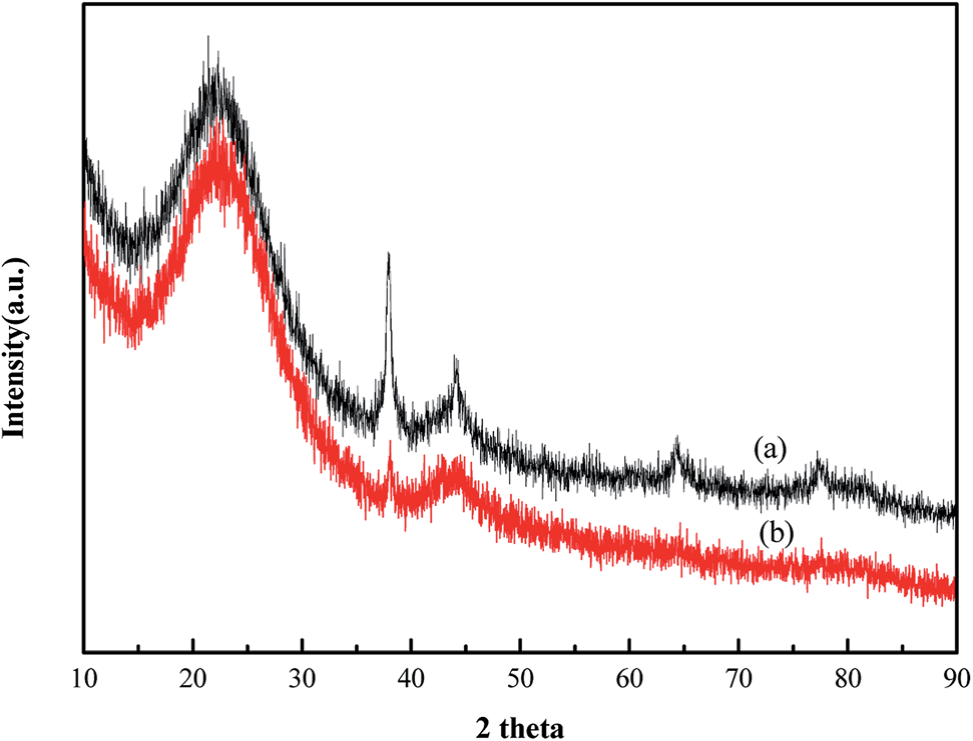
XRD patterns of the (a) 1% Au/AC and (b) 1% Au/HCS catalysts.

XRD characterization results of Au/HCS and Au/AC are shown in Fig. 2. Characteristic diffraction peaks appeared at 24.4°, which corresponded to the (002) diffraction of the carbon material in these two catalysts. In addition, the obvious diffraction peaks at 38.5°, 44.14°, 64.8°, and 78° corresponded to the (111), (200), (220), and (311) lattice planes of metallic Au^0^.^17^ This result shows that the Au nanoparticles were successfully deposited on the HCS and AC support. The Au (111) peak was used to calculate the particle size according to the Scherrer's equation. The Au nanoparticles size in Au/AC and Au/HCS catalysts were 13.7 nm and 6.3 nm, respectively. The results showed that the Au nanoparticles displayed a small particle size in the Au/HCS catalyst *versus* the Au/AC catalyst.

**Fig. 3:**
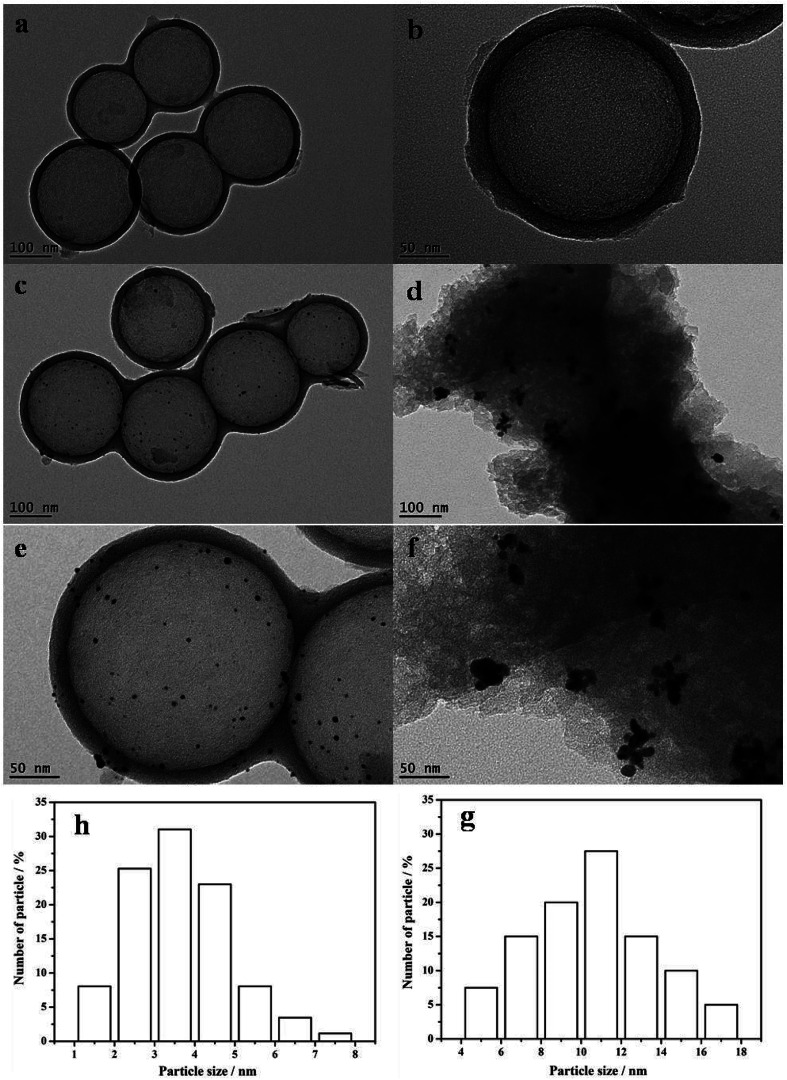
TEM data on the (a and b) HCS, (c and e) Au/HCS, (d and f) Au/AC. The figure also shows the catalysts and particle size histograms of (g) Au/HCS and (h) Au/C catalysts.

The TEM images shown in Fig. 3 represent the structures of the HCS, the Au/HCS catalyst, and the Au/AC catalyst. Fig. 3a and b shows that the contrast difference between the edge and the center of the spheres is obvious, indicating that the HCS was, indeed, hollow. The wall thickness was around 25 nm, and the diameter of the core is 190–220 nm. The TEM images of Au/HCS and Au/AC catalysts are shown in Fig. 3c–f. Fig. 3c and d shows that the Au nanoparticle dispersion is dispersed uniformly on the surface of the HCS, and some aggregation can be observed on AC. The average particle diameter of Au/HCS was 3.94 nm (Fig. 3h), and the average particle diameter of Au/AC was 11.43 nm (Fig. 3g). This result demonstrated that the HCS support was beneficial for controlling the size of Au nanoparticles and is consistent with the results.^18^

**Fig. 4:**
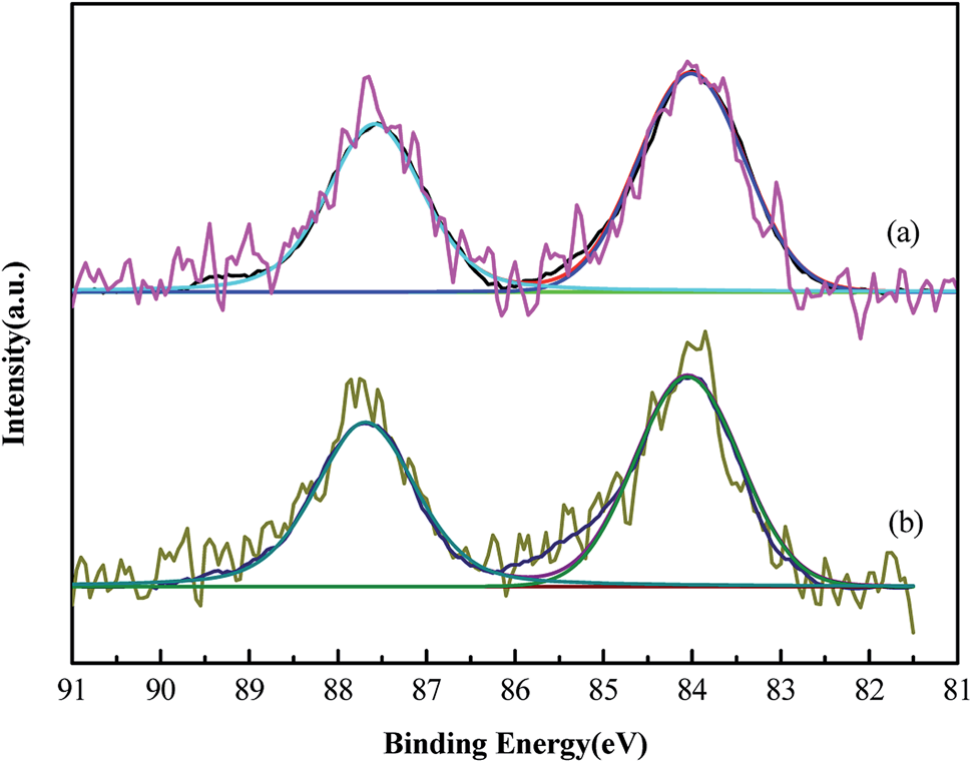
XPS spectra for the Au 4f of the (a) Au/AC and (b) Au/HCS catalysts.

XPS was performed because the catalytic activity of Au catalyst could be influenced by the gold valence (Fig. 4). The binding energy of Au 4f showed no change in the Au/AC and Au/HCS catalysts. The peaks at 84.1 eV and 87.8 eV were ascribed to the 4f_7/2_ and 4f_5/2_ peaks of metallic Au, respectively.^17,19^ No oxidative valences of Au(iii) and Au(i) were found in the XPS spectras of Au/AC and Au/HCS catalysts, indicating all oxidative Au was reduced by NaBH_4_ reduing agent in the catalyst preparing process. The results mean that the structure of the support had no influence on the reducing process of the Au precursor.

**Fig. 5:**
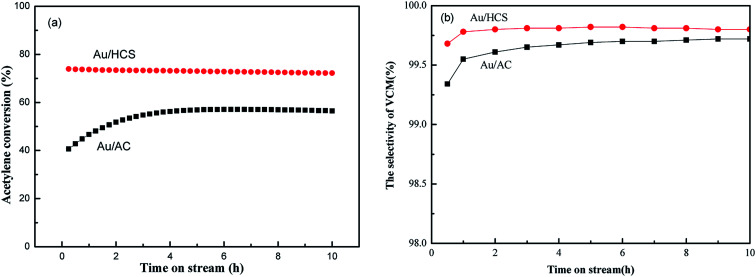
Conversion of acetylene (a) and selectivity of VCM (b) in acetylene hydrochlorination catalyzed by the Au/HCS and Au/AC catalysts (GHSV = 870 h^−1^, V_HCl_/V_C_2_H_2__ = 1.15, reaction temperature = 180 °C).

The catalytic activity of the Au/HCS and Au/AC catalysts were evaluated using a fixed-bed reactor containing 2 mL of the catalyst. The catalysts were evaluated under the following conditions: GHSV = 870 h^−1^, V_HCl_/V_C_2_H_2__ = 1.15, reaction temperature = 180 °C. The conversion of acetylene and selectivity of VCM during 10 h in the stream are shown in Fig. 5. Fig. 5b shows that the differences between HCS and AC supports have a very slight effect on the selectivity of VCM during acetylene hydrochlorination. The acetylene conversion of Au/AC catalyst was about 57%, being similar to our previous results of metallic Au nano-particles.^7^ However, acetylene conversion *via* the Au/HCS catalyst was much higher *versus* the Au/AC catalyst (Fig. 5a); the enhanced catalytic activity of Au/HCS may be assigned with the good dispersion of the Au nanoparticles.

**Fig. 6:**
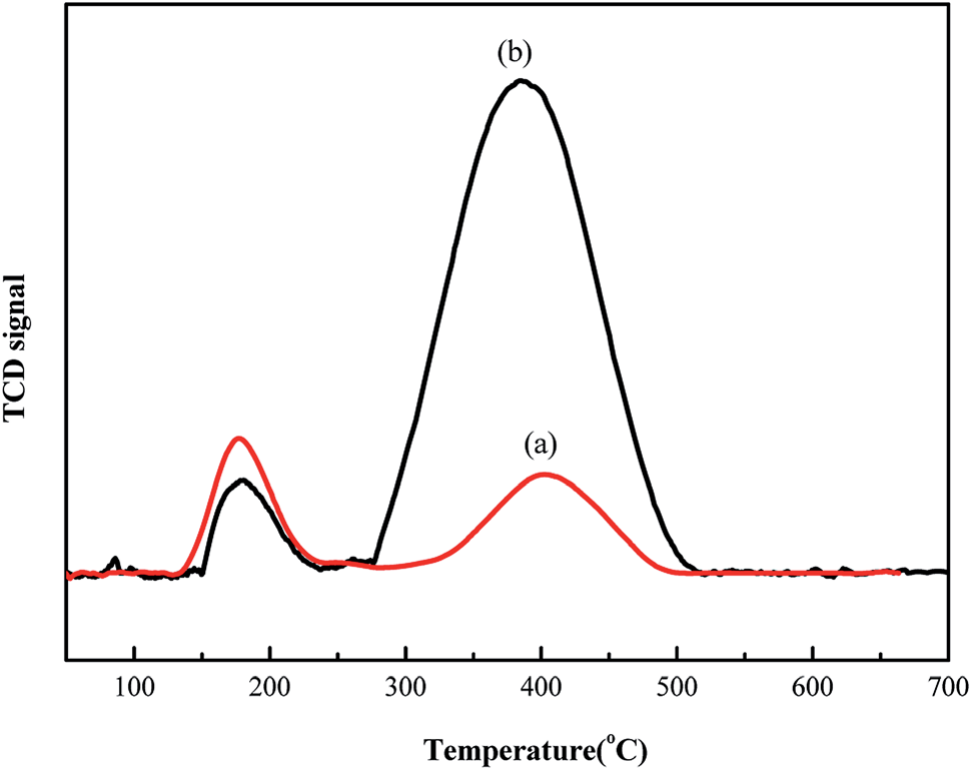
TPD-HCl profiles of the (a) Au/AC and (b) Au/HCS catalysts.

Next, TPD experiments were performed to analyze the adsorption strength of hydrogen chloride on the different supports. This study can help elucidate the mechanism underlying enhanced acetylene conversion *via* Au/HCS. Fig. 6 shows that the Au/AC catalyst has two desorption peaks at 195 °C and 410 °C. The desorption peaks in the Au/HCS catalyst were observed at 195 °C and 400 °C. It is obvious that the second peak of the Au/HCS catalyst was larger than that of the Au/AC catalyst. The adsorption of hydrogen chloride was the rate-controlling step in acetylene hydrochlorination.^20^ This phenomenon demonstrates that hydrogen chloride has stronger adsorption on the HCS during acetylene hydrochlorination than on active carbon. This might be the reason for the enhanced catalytic activity of Au/HCS.

**Fig. 7:**
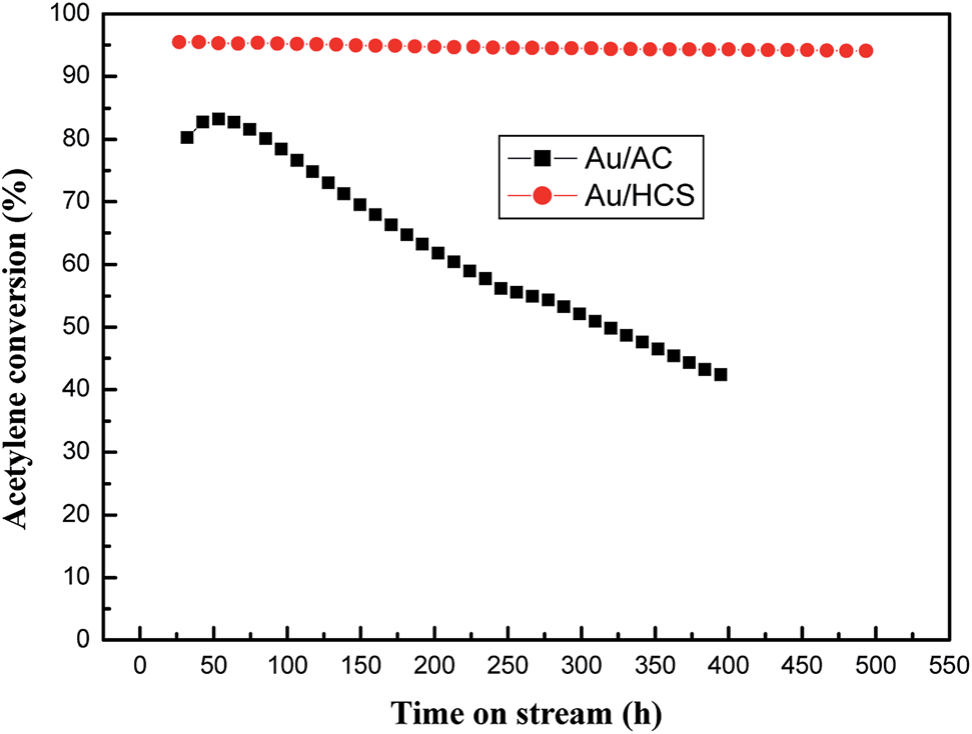
Long lifetime testing of Au/AC and Au/HCS catalysts (GHSV = 360 h^−1^, V_HCl_/V_C_2_H_2__ = 1.15, reaction temperature = 180 °C).

The most important parameter of the Au catalyst for acetylene hydrochlorination was its stability under harsh reaction conditions. Therefore, we compared the running life-times of Au/HCS and Au/AC at GHSV = 360 h^−1^, V_HCl_/V_C_2_H_2__ = 1.15, and reaction temperature = 180 °C. As shown in Fig. 7, the acetylene conversion of the Au/AC catalyst reduced from 82% to 43% after 400 h of running time. The Au/HCS catalyst exhibited excellent catalytic activity for acetylene hydrochlorination, and its acetylene conversion remained above 94% even after 500 h running time. Considered the harsh reaction conditions (GHSV = 360 h^−1^), the lifetime of Au/HCS under industrial condition (GHSV = 36 h^−1^) could be predicted to be higher than previous results reported in the literatures.^21,22^

**Fig. 8:**
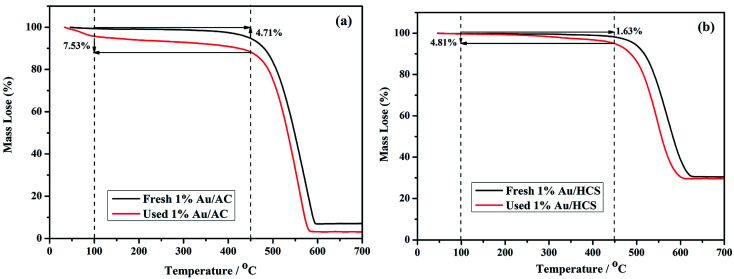
TGA curves of (a) Au/AC and (b) Au/HCS catalysts.

**Fig. 9:**
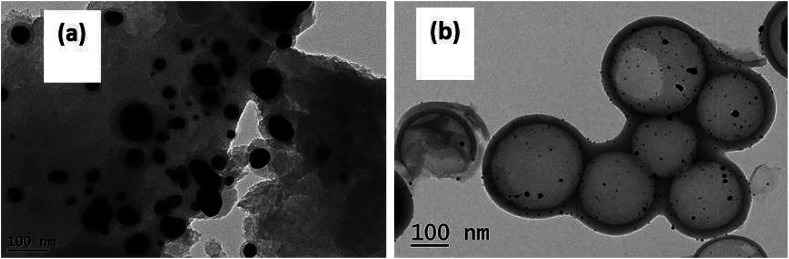
TEM images of the (a) spent Au/AC and (b) Au/HCS catalysts.

ICP-AES was used to test the actual Au loading. The actual Au loading was 0.89 wt% and 0.88 wt% in the fresh and spent Au/HCS catalyst, respectively. The results revealed that Au nano-particles did not leached in the acetylene hydrochlorination process. Oxidative Au reduction, aggregation of Au nanoparticles, and carbon deposition from acetylene polymerization might be the three reasons for Au deactivation.^7^ However, an oxidative Au reaction could be excluded because the XPS characterization only showed metallic Au(0) in Au/HCS and Au/AC catalysts. Next, TGA was used to characterize the carbon deposition of the catalysts, and TEM of the spent catalysts showed the dispersion of Au nanoparticles on HCS and AC support after the lifetime testing. These experiments could help further explain why the Au/HCS catalyst showed excellent stability for acetylene hydrochlorination. Fig. 8 indicates that the amount of carbon deposited on the Au/HCS catalyst is 1.73%, and that on the Au/AC is 4.71%. Carbon deposition resulted in clogged pores and reduced surface areas in the carrier—both of these decreased catalyst activity. The carbon cokes in the Au/HCS and Au/AC were minor, and thus carbon deposition was not the main reason for the quick deactivation of Au/AC catalyst. TEM was performed to determine whether the Au particle aggregation was the main reason for the deactivation rapid of the Au/AC catalyst (Fig. 9). Fig. 9b shows the spent Au/AC catalyst—the particle size of Au nanoparticles obviously increased after 400 h running, which indicates that the Au nanoparticles form aggregates during acetylene hydrochlorination. This indicates that aggregation of the Au nanoparticles was the main reason for the deactivation of the Au/AC catalyst. However, no obvious aggregation was seen in Au/HCS. The Au nanoparticles were still uniformly dispersed even after 500 h running, which means that the hollow structure of HCS could restrict the aggregation of Au nanoparticles during the acetylene hydrochlorination process. Mesopores originated from the decomposition of micelles could protect the interior Au nano-particles from aggregating with Au in other hollow carbon.^23^ In addition, the hollow shell of HCS can act as a barrier, which may be another reason for the weill dispersion of Au nanoparticles.^24^ The good dispersion of the Au nanoparticles due to the HCS structure may be the reason for the excellent stability of the Au/HCS catalyst.

## 4. Conclusions

The Au/HCS catalyst was successfully prepared and applied to the acetylene hydrochlorination reaction. *Versus* an AC support, the HCS support controlled the Au nanoparticle size to a mean of 3.94 nm, improved the dispersion of the Au particles, and inhibited agglomeration. Au/HCS displayed excellent catalytic activity and stability for acetylene hydrochlorination. The acetylene conversion of Au/HCS can be maintained above 92% even after 500 h lifetime running. The excellent catalytic performance of Au/HCS was attributed to the presence of the HCS support that limits the aggregation of Au nanoparticles.

## Conflicts of interest

There are no conflicts to declare.

## Supplementary Material
